# Epstein-Barr virus co-infection in a patient with dengue fever presenting with post-infectious cerebellitis: a case report

**DOI:** 10.1186/1752-1947-6-43

**Published:** 2012-01-30

**Authors:** Suneth Karunarathne, Yapa Udayakumara, Harshini Fernando

**Affiliations:** 1 Ward 49, National Hospital of Sri Lanka, Regent Street, Colombo 08, Sri Lanka

## Abstract

**Introduction:**

Post-infectious cerebellitis is an acute form of inflammatory encephalitis mainly limited to the cerebellum. It is commonly found in children, especially after viral infections such as Epstein-Barr virus. Post-infectious cerebellitis presents with acute onset dysarthria and ataxia. To the best of our knowledge, this is the first case report of post-infectious cerebellitis in a patient with both dengue and Epstein-Barr viral infection confirmed on serology.

**Case presentation:**

A 43-year-old Sri Lankan Sinhala man presented with an acute febrile illness associated with thrombocytopenia. While being managed as uncomplicated dengue fever, our patient developed dysarthria, ataxia and cerebellar signs. Our patient's infectious disease screen was positive for both dengue and Epstein-Barr specific immunoglobulin M. A cerebrospinal fluid analysis was suggestive of viral meningoencephalitis while cerebrospinal fluid serology was positive for dengue immunoglobulin G. T2-weighted magnetic resonance images were consistent with post-viral cerebellitis. The patient was given full supportive care and he made an uneventful complete recovery.

**Conclusion:**

There have been no previously reported cases of post-infectious cerebellitis associated with both Epstein-Barr and dengue viral infections confirmed by serology. Our patient's clinical features and findings on the imaging studies were consistent with post-viral cerebellitis. This report highlights the need to screen for other possible more common etiologies of a particular presentation before coming to a specific diagnosis based on initial findings. Uncomplicated cases of cerebellitis can be successfully managed with appropriate supportive measures with good prognosis, as in this case.

## Introduction

Post-infectious cerebellitis is an inflammatory disorder resulting in acute cerebellar dysfunction. Occurring most commonly in young children, it is an encephalitis largely restricted to the cerebellum [1].

Most of the reported cases of post-infective cerebellitis were due to chickenpox, Coxsackie virus, Epstein-Barr virus (EBV), Mycoplasma pneumonia and human immunodeficiency virus [2]. Despite an extensive literature survey, we could not find any reported cases of cerebellitis following dengue virus infection. There were no previously reported cases of acute post-infective cerebellitis in Sri Lanka due to any etiology.

## Case presentation

A 43-year-old previously healthy Sri Lankan Sinhala man was admitted with an acute febrile illness lasting for three days. He had high fever associated with severe myalgia, arthralgia and headache. He also had vomiting associated with a severe loss of appetite for two days. There was no photophobia. He did not complain of cough, chest pain or shortness of breath. There was no dysuria, increased frequency of urination, loin pain, diarrhea or abdominal pain. He did not have any bleeding manifestations. He had no history of skin rash or joint swelling. He had not noticed any significant reduction of urine output and his urine color was normal.

On examination, our patient was febrile and appeared ill. He was not icteric and not pale. There was no cervical lymphadenopathy. There was a mild conjunctival injection but no conjunctival hemorrhages. There were no peripheral stigmata to suggest bacterial endocarditis. Neck stiffness and Kernig's sign were absent. There were no features of joint inflammation but his muscles were mildly tender. No skin rashes or eschars were observed.

A cardiovascular examination found our patient to be tachycardic with a regular pulse rate of 104 beats per minute. His jugular venous pressure was not elevated. His blood pressure was 110/68 mmHg with no postural decline. His heart was in dual rhythm and there were no murmurs. His apex beat was not deviated.

He was not dyspnoic and had a respiratory rate of 16 breaths per minute. His breath sounds were symmetrically vesicular and there was no evidence of consolidation. No rhonchi or crepitations were observed.

His abdomen was soft to superficial palpation. There were no abnormal masses on deep palpation and his liver and spleen were not palpable, nor were there any ballotable masses. There was no free fluid.

A nervous system examination revealed an alert and conscious middle-aged man who was mildly distressed due to headache and myalgia. A cranial nerve examination and neurological examination did not reveal any abnormality and he had a normal gait.

Basic laboratory investigations were sent and our patient was initially managed as having an unspecified viral fever pending results of the investigations. A full blood count revealed his hemoglobin level to be 13.4 g/dL, his white blood cell count (WBC) 4400/μL (neutrophils, 76%; lymphocytes, 24%) and a platelet count of 48,000/μL. His serum creatinine was 83 μmol/L, serum sodium was 141 mmol/L and potassium was 3.7 mmol/L. His liver enzyme results were as follows: aspartate transaminase, 153 U/L, alanine transaminase, 207 U/L; and alkaline phosphatase, 387 U/L. His total protein level was 70 g/dL with albumin of 43 g/dL. His international normalized ratio was 0.97 and activated partial thromboplastin time 32 seconds.

The initial investigations were compatible with dengue fever. Blood was taken to screen for the dengue antibody and blood cultures were sent. His fluid intake was maintained according to the national guidelines on dengue fever, as there was a widespread dengue epidemic with hundreds of patients getting admitted daily. His fever was treated symptomatically with paracetamol.

His fever settled on the fourth day of fever and our patient was symptomatically better. However, he developed mild slurring of speech six days after the onset of fever. On the seventh day, he had marked slurring of speech and he found it difficult to walk due to severe unsteadiness. An examination revealed a bilaterally impaired finger-to-nose test and heel-to-shin test. He had dysdiadochokinesia and an ataxic gait. There was no nystagmus. An examination of the fundus of his eye did not reveal any abnormality. An examination of his cranial nerves continued to be normal. There were no peripheral sensory or motor deficits. His deep tendon reflexes and plantar reflexes remained normal.

**Figure 1:**
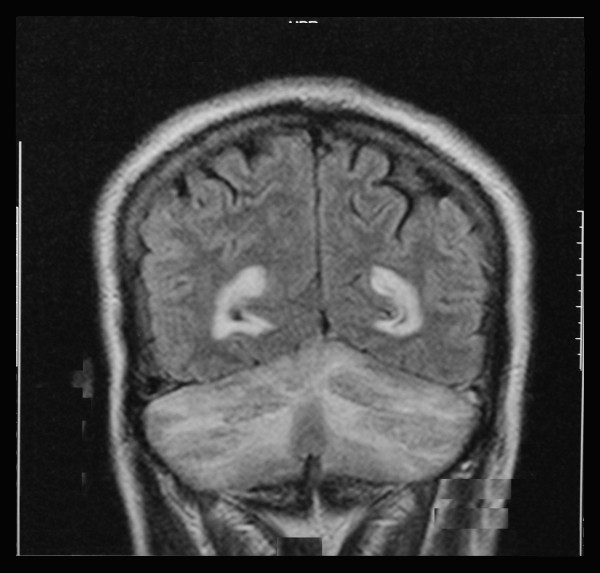
**Coronal magnetic resonance imaging showing hyperintense areas in his cerebellar white matter in a T2-weighted sequence**.

**Figure 2:**
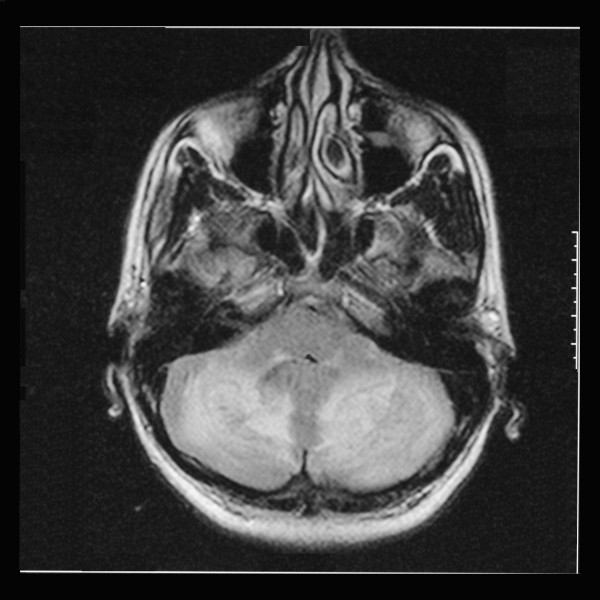
**Axial magnetic resonance imaging showing hyperintense areas in his cerebellar white matter and ventricular enhancement in a T2-weighted sequence**.

Due to the sudden onset of bilateral cerebellar signs, an urgent non contrast computed tomography scan was performed. There were bilateral hypodense areas in his cerebellum but his ventricles and cerebral hemispheres appeared normal. A magnetic resonance imaging (MRI) scan of his head was performed, which revealed bilateral diffuse hyperintense areas in his cerebellar hemispheres spreading across the vermis (Figures 1 and 2). The appearance in the MRI scan was compatible with acute postinfective cerebellitis. Serological studies were performed for the possible viral etiologies that can cause cerebellitis. Hepatitis B and C serology, herpes simplex virus serology and Japanese encephalitis immunoglobulin M were negative. An enzyme linked immunosorbent assay (ELISA) for human immunodeficiency virus was negative. Dengue virus immunoglobulin M (IgM) and immunoglobulin G (IgG) were both detected in our patient's serum on day six after the onset of the symptoms. EBV IgM antibody was detected in his blood by ELISA on the eighth day of illness. Serological studies for *Salmonella *typhi and paratyphi were negative. A cerebrospinal fluid (CSF) analysis showed a WBC of 10 cells/μL (lymphocytes, nine cells and neutrophils, one cell), red blood cells 1 cell/μL, proteins 0.88 g/dL and sugar 3.1 mmol/L with a random blood sugar of 5.4 mol/L, all of which were compatible with viral central nervous system infection. Cerebrospinal fluid cultures were negative and viral studies were negative for Herpes simplex virus, EBV antibody and Japanese encephalitis virus. Dengue IgG was detected in his CSF while IgM was negative. Dengue virus non-structural protein 1 antigen was not detected in his CSF.

Dependent on the clinical findings and serology, a diagnosis of post-infectious cerebellitis was made. Our patient was managed with maximum supportive care and physiotherapy combined with gait training. Speech therapy was also commenced. At discharge two weeks after the onset of symptoms, our patient was able to walk without support and his speech was markedly improved. His platelet count, WBC and differential counts were within normal limits at the time of discharge.

## Discussion

Since our patient had serological evidence of both recent dengue and EBV infections we could not attribute his clinical picture to a single viral agent. However, the clinical picture was more in favour of dengue fever rather than that of infectious mononucleosis. Usually, post-viral cerebellitis follows a benign course without life-threatening complications [3]. Acute hydrocephalus is one of the more serious among reported complications [4].

Acute cerebellitis when associated with cerebellar swelling, hydrocephalus and brainstem compression can be life-threatening, although these complications are rarely observed [5]. MRI shows hyperintense signals of cerebellar white matter in T2-weighted sequences, which is a strong indication of a diagnosis of acute cerebellitis (Figures 1 and 2). C-shaped cerebellar white matter T2 hyperintensity demonstrated in post-infectious cerebellitis is caused by cerebellar white matter vasogenic edema [6]. Resolution of the hyperintense areas in the cerebellar cortex can be associated with recovery from the clinical manifestations, although mild cerebellar atrophy may be seen in follow up MRI scans [7].

Although commonly due to viruses, bacterial infections have also been associated with cerebellitis, including *Borrelia burgdorferi *(Lyme disease), *Mycoplasma pneumoniae, Legionella *and *Coxiella burnettii *(Q fever). In addition, cerebellitis may follow immunizations, such as hepatitis, smallpox and measles vaccination. In many cases, however, the precise causative agent is not isolated [1]. In uncomplicated cases of cerebellitis treatment is usually supportive, as was the case in our patient.

## Conclusion

To the best of our knowledge, there have been no previously reported cases of cerebellitis associated with dengue viral infection. Despite having clinical and laboratory evidence suggestive of dengue, other possible etiological agents for post-infectious cerebellitis were sought as this was an unusual presentation of dengue. Besides describing interesting MRI features of post-infectious cerebellitis, this case illustrates the importance of having an open mind when an unusual presentation of a known disease is investigated. It therefore improves our understanding of clinical and laboratory features of post-infectious cerebellitis and also provides insight into unusual clinical presentations in general.

## Competing interests

The authors declare that they have no competing interests.

## Consent

Written informed consent was obtained from the patient for publication of this case report and any accompanying images. A copy of the written consent is available for review by the Editor-in-Chief of this journal

## Authors' contributions

SK, YU and HF were involved in the management of the patient. SK prepared the manuscript. YU and HF provided valuable input during the preparation of the manuscript. All authors read and approved the final manuscript.

## References

[B1] No authors listed Case records of the Massachusetts General Hospital. Weekly clinicopathological exercises. Case 38-1996. An 18-year-old man with severe headache, pleocytosis, and ataxia N Engl J Med 1996 335 24 1829 1834 10.1056/NEJM199612123352408 8943166

[B2] Teive HA Zavala JA Iwamoto FM Bertucci-Filho D Werneck LC Acute cerebellitis caused by Epstein-Barr virus: case report Arq Neuropsiquiatr 2001 59 3A 616 618 10.1590/S0004-282X2001000400027 11588648

[B3] Connolly AM Dodson WE Prensky AL Rust RS Course and outcome of acute cerebellar ataxia Ann Neurol 1994 35 673 679 10.1002/ana.410350607 8210223

[B4] Melaiki TB Thabet F Anjum SB Amin A Acute cerebellitis with hydrocephalus and brainstem compression Arch Dis Child 2007 92 38 10.1136/adc.2006.105874 17185444 PMC2083131

[B5] Levy EI Harris AE Omalu RL Hamilton RL Branstetter BF Pollack IF Sudden death from fulminant cerebellitis Pediatr Neurosurg 2001 35 24 28 10.1159/000050381 11490187

[B6] Montenegro MA Santos SL Li LM Cendes F Neuroimaging of acute cerebellitis J Neuroimag 2002 12 72 74 10.1111/j.1552-6569.2002.tb00095.x 11826604

[B7] Dogulu F Onk A Kaymaz M Kardes O Baykaner MK Acute cerebellitis with hydrocephalus Neurology 2003 60 1717 12771279 10.1212/01.wnl.0000064168.64413.ff

